# Supplementary material for: A Critical View of the Application of the APEX Software (Aqueous Photochemistry of Environmentally-Occurring Xenobiotics) to Predict Photoreaction Kinetics in Surface Freshwaters
Source: Molecules. 2019 Dec 18;25(1):9. doi: 10.3390/molecules25010009 (PMC7017383; doi:10.3390/molecules25010009)
Supplement: Supplementary file 1 [file molecules-25-00009-s001.zip › 3.2.4_gcc-4.4.0.zip!/3.2.4_gcc-4.4.0/3.2.4_gcc-4.4.0/doc/octave/html/interpreter/Comments.html]

Comments - Untitled


Previous: Executable Octave Programs,
Up: Getting Started


---

### 2.7 Comments in Octave Programs

A comment is some text that is included in a program for the sake
of human readers, and which is NOT an executable part of the program.
Comments can explain what the program does, and how it works. Nearly all
programming languages have provisions for comments, because programs are
typically hard to understand without them.

- Single Line Comments- Block Comments- Comments and the Help System
